# Extracorporeal decarboxylation in patients with severe traumatic brain injury and ARDS enables effective control of intracranial pressure

**DOI:** 10.1186/s13054-015-1088-1

**Published:** 2015-10-30

**Authors:** Christopher Munoz-Bendix, Kerim Beseoglu, Rainer Kram

**Affiliations:** Department of Neurosurgery, Medical Faculty, Heinrich-Heine-University Düsseldorf, Moorenstrasse 5, 40225 Düsseldorf, Germany; Department of Anesthesiology, Medical Faculty, Heinrich-Heine-University Düsseldorf, Moorenstrasse 5, 40225 Düsseldorf, Germany

## Abstract

**Introduction:**

Acute respiratory distress syndrome (ARDS) with concomitant impairment of oxygenation and decarboxylation represents a complex problem in patients with increased intracranial pressure (ICP). Permissive hypercapnia is not an option to obtain and maintain lung-protective ventilation in the presence of elevated ICP. Pumpless extracorporeal lung assist (pECLA) devices (iLA Membrane Ventilator; Novalung, Heilbronn, Germany) can improve decarboxylation without aggravation associated with invasive ventilation. In this pilot series, we analyzed the safety and efficacy of pECLA in patients with ARDS and elevated ICP after severe traumatic brain injury (TBI).

**Methods:**

The medical records of ten patients (eight male, two female) with severe ARDS and severe TBI concurrently managed with external ventricular drainage in the neurointensive care unit (NICU) were retrospectively analyzed. The effect of pECLA on enabling lung-protective ventilation was evaluated using the difference between plateau pressure and positive end-expiratory pressure, defined as driving pressure (ΔP), during the 3 days preceding the implant of pECLA devices until 3 days afterward. The ICP threshold was set at 20 mmHg. To evaluate effects on ICP, the volume of daily cerebrospinal fluid (CSF) drainage needed to maintain the set ICP threshold was compared pre- and postimplant.

**Results:**

The ΔP values after pECLA implantation decreased from a mean 17.1 ± 0.7 cm/H_2_O to 11.9±0.5 cm/H_2_O (*p* = 0.011). In spite of this improved lung-protective ventilation, carbon dioxide pressure decreased from 46.6 ± 3.9 mmHg to 39.7 ± 3.5 mmHg (*p* = 0.005). The volume of daily CSF drainage needed to maintain ICP at 20 mmHg decreased significantly from 141.5 ± 103.5 ml to 62.2 ± 68.1 ml (*p* = 0.037).

**Conclusions:**

For selected patients with concomitant severe TBI and ARDS, the application of pECLA is safe and effective. pECLA devices improve decarboxylation, thus enabling lung-protective ventilation. At the same time, potentially detrimental hypercapnia that may increase ICP is avoided. Larger prospective trials are warranted to further elucidate application of pECLA devices in NICU patients.

## Introduction

*Acute respiratory distress syndrome* (ARDS), according to the recent Berlin definition, is defined as impaired oxygenation caused by acute, diffuse, inflammatory lung injury [1]. Though inflammatory in nature, ARDS is often triggered by aspiration and traumatic lung injury in the context of patients with severe brain injuries and impaired consciousness. Thus, it is not uncommon in neurocritically ill patients, and it is a predictor of in-hospital mortality [2]. The management of ARDS comprises mechanical ventilation with low tidal volume (V_T_), a plateau pressure (P_plat_) <30 cm/H_2_O, and positive end-expiratory pressure (PEEP) adjusted to the fraction of inspired oxygen (FiO_2_) [3]. This lung-protective ventilation (LPV) concept may cause hypercapnia, which is tolerated as long as oxygenation is adequate and pH remains >7.1 [4, 5]. However, hypercapnia impairs cerebral hemodynamics and autoregulation of cerebral blood flow per se. In patients with traumatic brain injury (TBI), the latter can aggravate a preexisting alteration of cerebral autoregulation [6–9]. This may result in an increase of intracranial pressure (ICP), with potentially deleterious effects for the injured brain. The treatment of patients with concomitant TBI and ARDS is challenging because the management of ICP requires avoiding permissive hypercapnia. Therefore, alternative interventions are required to treat both pathologies appropriately. Pumpless extracorporeal lung assist (pECLA) devices can improve decarboxylation, thus enabling LPV while stabilizing carbon dioxide pressure (pCO_2_) at tolerable levels for patients requiring rigorous ICP management [10–12].

In this retrospective patient cohort, we endeavored to evaluate the use of a pECLA device (iLA Membrane Ventilator; Novalung, Heilbronn, Germany) in patients with severe brain injury and concomitant ARDS in a neurointensive care unit (NICU).

## Methods

### Patient selection

We retrospectively analyzed the medical records of ten patients (eight male, two female) treated in our NICU between 2011 and 2014. The data collection and analysis and publication of the results were approved by the local ethics committee (ethics committee of Heinrich Heine University Düsseldorf, Chairman Prof. Kröncke, study 4516). No consent from the patients for inclusion of their data in this analysis was necessary, so it was not obtained. Individual details potentially jeopardizing patients’ anonymity are omitted.

The decision regarding pECLA treatment for patients with TBI (Abbreviated Injury Scale score >3) was based on the clinical constellation of mild to moderate ARDS refractory to conservative measures according to the Berlin definition with insufficient oxygenation despite LPV and poor or unapparent decarboxylation (pCO_2_ > 45 mmHg) with concomitant elevated ICP >20 mmHg measured continuously with an external ventricular catheter (EVD) [1, 13, 14]. Conservative interventions used to control elevated ICP included deep sedation without myorelaxation, prone positioning with 30-degree elevated head rest, and drainage of cerebrospinal fluid (CSF) via the EVD.

Ventilator therapy focused on achieving a low V_T_ of 6 ml/kg of predicted body weight and avoiding excessive ventilation pressure >30 cm/H_2_O. We aimed at normalizing pCO_2_ levels between 35 mmHg and 45 mmHg. Clinical interventions comprised an increase in respiratory rate to enable adequate ventilation as long as individual expiratory time remained sufficient. When this yielded no satisfactory effect on pCO_2_, we increased the driving pressure (ΔP) by increasing the P_plat_.

### Application of extracorporeal carbon dioxide elimination

The use of extracorporeal lung assist is an established supportive therapeutic strategy in ARDS [15]. We used the iLA Membrane Ventilator [11]. Under ultrasonographic control, a 15-French, 90-mm arterial cannula was inserted into a femoral artery and a second cannula (17 French, 140 mm) was placed in the opposite femoral vein using the Seldinger technique. Driving force for the perfusion of the membrane system is the patients’ arteriovenous pressure difference. Thus, systemic blood pressure must be sufficient to enable blood to flow through the pECLA system at 1.3–1.7 L/min. Through an oxygen supply line connected to the inflow site of the membrane oxygenator, a continuous oxygen flow of 6–10 L/min is established to achieve carbon dioxide extraction from the blood.

Partial thromboplastin time (PTT) >50 s is recommended to prevent blood clotting in the system; however, all surfaces in contact with circulating blood are heparin-coated to reduce the risk of coagulation. All patients received continuous intravenous heparin infusions with a target PTT ranging from 50 to 60 s. The effect of anticoagulation was controlled every 12 h, and the heparin infusion was adjusted accordingly.

pECLA device was discontinued when decarboxylation improved and ICP was stable enough to allow the reduction of sedation and spontaneous breathing. Subsequently, the continuous oxygen flow of the membrane oxygenator was diminished to reduce carbon dioxide extraction, and the pECLA device was removed when pCO_2_ remained stable.

### Statistical analysis

Ventilation parameters [ΔP, P_plat_, mean ventilation pressure (P_mean_), PEEP, V_T_, pCO_2_, pH] from 3 days preceding pECLA device implant until 3 days afterward were compared. All ventilation-related variables, such as V_T_ in milliliters, P_plat_ in cm/H_2_O, P_mean_ in cm/H_2_O, PEEP in cm/H_2_O, blood pH level, and ΔP in cm/H_2_O (defined as P_plat_ minus PEEP) were recorded half-hourly [16]. Additionally, the ratio of oxygen pressure (pO_2_) to FiO_2_ (pO_2_/FiO_2_) was calculated every 4–6 h based on arterial blood gas analysis and the ventilator setting. Mean arterial pressure (MAP) was recorded continuously for each patient. To calculate mean values, a single reading every 30 min was taken. Last, the fluid balance of each patient was recorded daily for a 24-h period.

To quantify the effects of ICP management, the volume of daily CSF drainage was compared before and after the pECLA device was implanted. The amount of drained CSF necessary to maintain the set ICP threshold of 20 mmHg was recorded every 24 h.

Statistical analysis was performed using IBM SPSS version 15.1.1 software (IBM, Armonk, NY, USA). To identify differences between relevant parameters before and after the pECLA device was implanted, the Wilcoxon signed-rank test for related samples was applied. Significance was accepted at a level of *p* < 0.05. Depending on the variable, means with standard deviations or medians with interquartile ranges (IQRs) are given.

## Results

The mean patient age was 40.2 ± 16.5 years, and all patients developed ARDS between 2 and 7 days after admission (median 4.5 days; IQR 3.3–5.8 days). The pECLA device was implanted a median of 1.5 (IQR 1–2) days after ARDS was diagnosed and was removed after a median of 8 days (IQR 7–9 days). The relevant patient characteristics and data are shown in Table 1.

**Table 1:** Patient characteristics

Patient	Age (yr)	ARDS diagnosis (days after admission)	pECLA device implant after ARDS diagnosis (days)	pECLA device treatment (days)
1	39	7	2	12
2^a^	61	6	2	7
3	40	4	1	5
4^a^	35	2	2	7
5	13	5	1	12
6	53	3	1	4 (6)^b^
7	53	5	2	8
8	50	6	1	8
9	53	4	2	8
10	50	3	1	9

^a^Patient died within 30 days after admission

^b^Patient was treated after day 4 with venovenous extracorporeal membrane oxygenation because of acute decreasing oxygenation

**Fig. 1:**
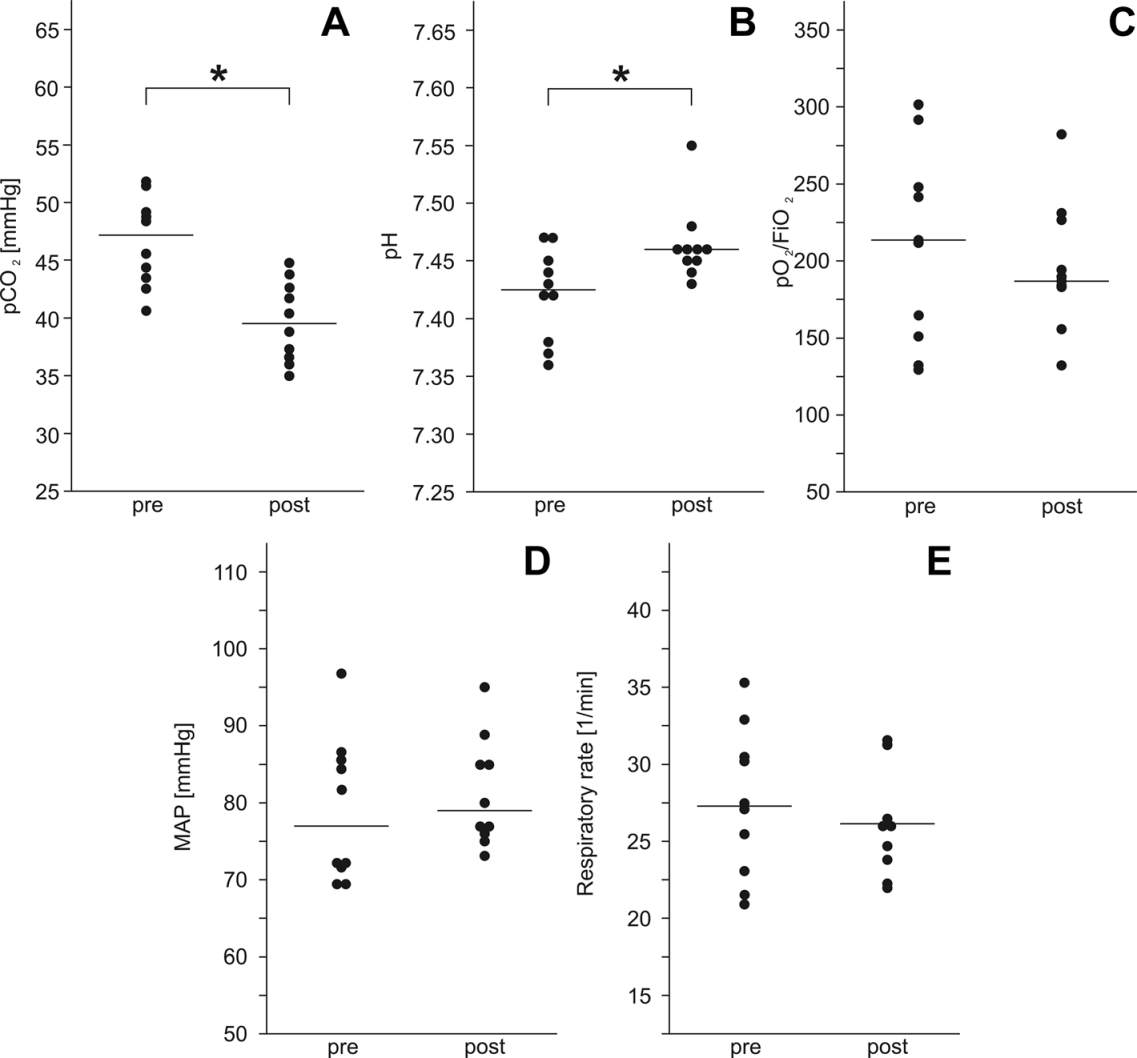
Changes in carbon dioxide pressure (pCO_2_), pH, mean arterial pressure (MAP), oxygen pressure to fraction of inspired oxygen (pO_2_/FiO_2_) ratio, and respiratory rate after implant of the pumpless extracorporeal lung assist device. **a** After implant (post) of the pumpless extracorporeal lung assist (pECLA) device, pCO_2_ was significantly reduced compared with before (pre)implant. **b** Simultaneously, pH increased significantly postimplant as compared with preimplant. The pO_2_/FiO_2_ ratio (**c**), MAP (**d**), and respiratory rate (**e**) remained unchanged before and after implant of the pECLA device.

Implant of the pECLA device facilitated a significant decrease of pCO_2_ from 46.6 ± 3.9 mmHg to 39.7 ± 3.5 mmHg (*p* = 0.005), and pH rose from 7.42 ± 0.04 to 7.46 ± 0.03 (*p* = 0.021) (Fig. 1a, b). The pO_2_/FiO_2_ ratio remained unchanged after implant of the pECLA device (Fig. 1c). MAP was kept stable over the treatment period (Fig. 1d), and fluid was kept at a positive balance of 1500–2000 ml daily to stabilize patients’ hemodynamics. Respiratory rate showed no significant change, but a slight decrease was seen (Fig. 1e).

**Fig. 2:**
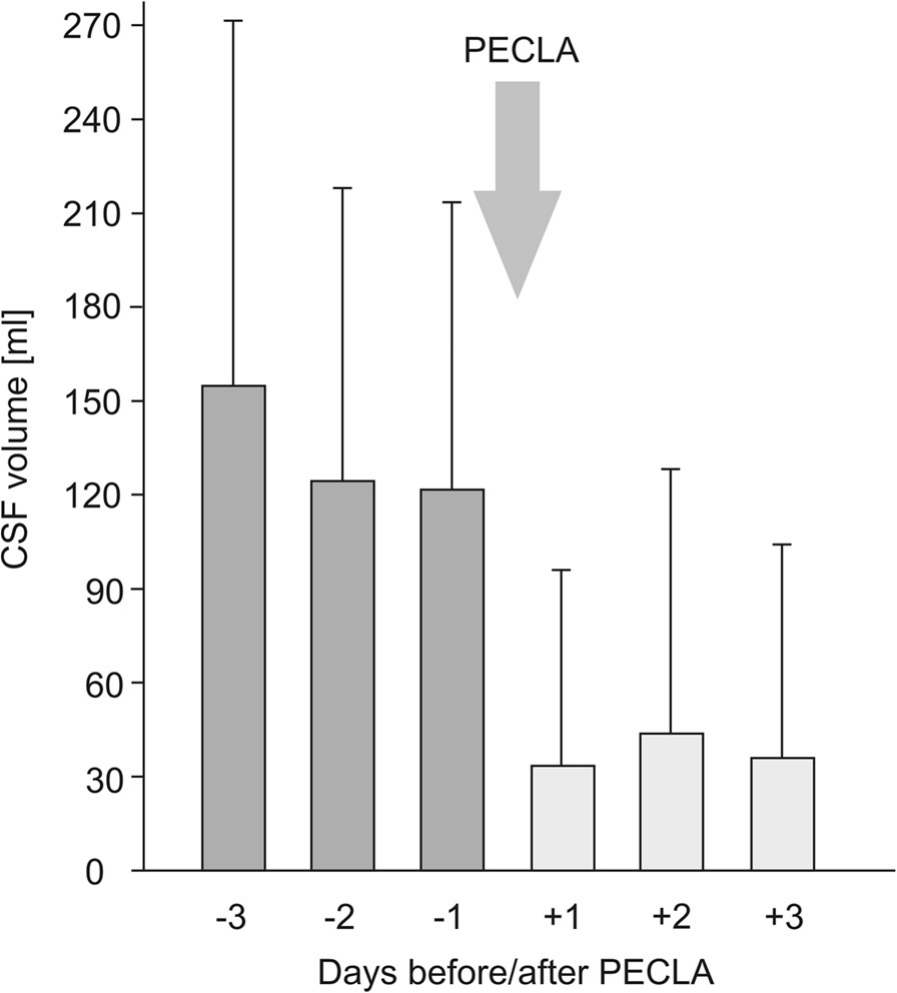
Daily cerebrospinal fluid (CSF) drainage. The mean volume of drained CSF per 24 h from 3 days preceding implant of the pumpless extracorporeal lung assist (pECLA) device until 3 days afterward is shown. The *arrow* marks the time of implant of the pECLA device, and the *whiskers* represent the standard deviation

The mean volume of daily CSF drainage needed to maintain ICP <20 mmHg decreased significantly from 141.5 ± 103.5 ml to 62.2 ± 68.1 ml (*p* = 0.037) (Fig. 2).

**Fig. 3:**
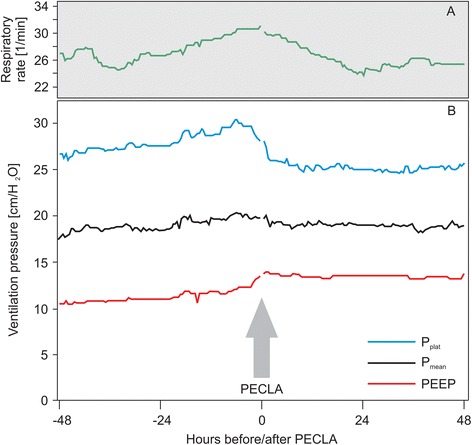
Changes in plateau pressure (P_plat_), mean pressure (P_mean_), and positive end-expiratory pressure (PEEP) after implant of the pumpless extracorporeal lung assist device. **a** The respiratory rate was gradually increased before pumpless extracorporeal lung assist (pECLA) device implant, corresponding to the increase of airway pressure, and then decreased afterward. **b** Ventilation parameters for P_plat_, P_mean_, and PEEP every 30 min are illustrated. After implant of the pECLA device (*arrow*), P_plat_ was significantly reduced (*p* < 0.001), whereas PEEP was increased significantly (*p* < 0.001). P_mean_ remained unchanged

Mean P_plat_ was significantly reduced [28.0 ± 1.0 cm/H_2_O to 25.2 ± 0.5 cm/H_2_O, 95 % (CI) of the difference 2.6–3.1 cm/H_2_O; *p* < 0.001], and PEEP was increased significantly (11.5 ± 0.7 cm/H_2_O to 13.7 ± 0.2 cm/H_2_O, 95 % CI of the difference 2.1–2.4 cm/H_2_O; *p* < 0.001). Both interventions caused a decrease of ΔP from a mean of 17.1 ± 0.7 cm/H_2_O to 11.9 ± 0.5 cm/H_2_O (*p* = 0.011), whereas P_mean_ remained unchanged (19.3 ± 0.7 cm/H_2_O to 19.2 ± 0.3 cm/H_2_O; *p* = 0.726) (Fig. 3).

In our cohort, one patient developed right heart failure 4 days after implant of the pECLA device. The pECLA device was immediately discontinued, and the patient was treated with a venovenous extracorporeal membrane oxygenation (ECMO) system. She recovered without sequelae and was discharged from the intensive care unit after 20 days.

## Discussion

In this report of a patient cohort with ARDS and TBI treated with implant of a pECLA device, we describe several important findings. First, pECLA is a safe and effective tool to enable LPV in patients with ARDS in the presence of severe brain injury. Second, in the critical situation of elevated ICP and concomitant demand for adjusted ventilation, the use of pECLA enables better ICP control under optimized ventilator settings. Third, the better control of ICP, as reflected in the reduced necessity to drain CSF, results from optimized pCO_2_ levels and reduced ΔP and occurs regardless of P_mean_.

Though most patients with TBI die as a result of the underlying pathology or secondary complications rather than because of respiratory failure per se, ARDS contributes significantly to poor outcome and increased risk of death in these patients [2, 17]. The fundamental treatment approach in ARDS is to achieve adequate oxygenation and decarboxylation by optimizing mechanical ventilation. However, mechanical ventilation can induce lung injury and thus exacerbate the pathology itself, and it can also contribute to dysfunction of other organs [18, 19]. In order to reduce alveolar overdistension, which is the most frequent ventilator-induced lung injury, the use of low V_T_ ventilation (i.e., LPV) is the preferred approach [3, 20]. LPV can cause hypercapnia, which is usually well-tolerated and thus referred to as *permissive hypercapnia* [21]. In addition to avoiding high peak inspiratory pressures, the concept of LPV is to use high PEEP to improve oxygenation and reduce or prevent atelectasis [22, 23].

In neurosurgical patients with TBI, permissive hypercapnia cannot be tolerated easily. Hypercapnia increases cerebral blood flow by cerebral vasodilation. More specifically, with increasing pCO_2_, the autoregulatory pressure range is decreased and, depending on the pCO_2_ level, cerebral autoregulation is significantly impaired [3, 8, 9]. In severe hypercapnia with pCO_2_ > 70 mmHg, the pressure–flow relationship can approximate a linear relationship, as demonstrated in animal experiments [24]. Thus, hypercapnia in these vulnerable patients can aggravate blood flow dysregulation and cause detrimental increases in ICP. Extracorporal carbon dioxide removal using pECLA is effective in patients in a tenuous situation of severe lung and brain injury [10, 25]. pCO_2_ is effectively reduced to normal levels and can be controlled by adjusting the oxygen sweep flow to avoid unwanted hypocapnia. Normalization of pCO_2_ is accompanied by increased pH and avoidance of respiratory acidosis. This yields a significant reduction in drained CSF as a surrogate for improved ICP control. Interestingly, mean airway pressure does not contribute to this effect. After pECLA device implant, inspiratory pressure is decreased, but PEEP is increased, to improve oxygenation. As a consequence, mean airway pressure does not change significantly. Therefore, improved ICP control can be attributed predominantly to the normalization of pCO_2_. In ARDS, the combination of low V_T_ and higher PEEP is superior to low V_T_ with lower PEEP [26]. Though application of PEEP in patients with brain injury remains controversial, a judicious adaption of PEEP to the demand in patients with lung injury is feasible [27]. High peak pressures do have a deleterious effect on cerebral hemodynamics, however, and can be avoided in this setting [7].

An important limitation of our analysis is its retrospective nature; thus, residual confounding parameters might have been missed. In addition, the number of patients is too small to allow generalizability of the results or to provide any meaningful outcome data. However, the main aim of this study was to provide data on the safety and efficacy of this intervention.

In this context, it is important to recognize that, for pECLA treatment, systemic anticoagulation to avoid clotting of the filter membrane is recommended. In patients with brain injury, this may increase the risk of decompensating intracerebral hematomas. However, in our series, no complications (cerebral or extracerebral) attributable to anticoagulation were seen. Besides, owing to heparin-coating of the extracorporeal circuit, low-dose systemic anticoagulation is sufficient to avoid clotting of the extracorporeal circuit, thereby minimizing the risk of bleeding complications. In patients with extraordinarily high risk of bleeding, even a complete abdication of anticoagulation in venovenous ECMO was shown to be safe and feasible [28]. Also, elevated ICP and CSF production are multifactorial processes, and in the small, retrospective setting of the present study, confounding hemodynamic or metabolic effects contributing to the reduction in CSF drainage with the use of pECLA may have been missed.

Owing to the relevant shunt volume of approximately 1500 ml of blood per minute over the extracorporeal circuit, a relevant increase in right heart preload occurs. This may have contributed to the right heart failure in one of our patients. This patient had no known preexisting cardiac disease; however, the patient was obese (body mass index 33 kg/m^2^), which might have contributed to reduced right ventricular capacity.

## Conclusions

For selected patients with concomitant severe brain and lung injury, the application of pECLA is effective to avoid hypercapnia and respiratory acidosis, thus enabling application of LPV guidelines as well as effective ICP control. Large prospective trials are necessary to further elucidate the application of pECLA devices and the influence of different ventilation pressures on ICP and outcome in NICU patients.

## Key messages

- pECLA is a safe and effective tool to enable LPV in patients with ARDS and TBI.
- pECLA effectively reduces pCO_2_ and normalizes pH in patients vulnerable to hypercapnia.
- pECLA enables better ICP control under optimized ventilator settings than without extracorporeal decarboxylation.

## Abbreviations

ARDS: Acute respiratory distress syndrome
CI: Confidence interval
CSF: Cerebrospinal fluid
ECMO: Extracorporeal membrane oxygenation
EVD: External ventricular catheter
FiO_2_: Fraction of inspired oxygen
ICP: Intracranial pressure
IQR: Interquartile range
LPV: Lung-protective ventilation
MAP: Mean arterial pressure
NICU: Neurointensive care unit
ΔP: Driving pressure
pCO_2_: Carbon dioxide pressure
pECLA: Pumpless extracorporeal lung assist
PEEP: Positive end-expiratory pressure
P_mean_: Mean ventilation pressure
P_plat_: Plateau pressure
PTT: Partial thromboplastin time
TBI: Traumatic brain injury
V_T_: Tidal volume
